# Clozapine and Sweet's syndrome: case report

**DOI:** 10.1192/bjo.2023.513

**Published:** 2023-09-04

**Authors:** Apphia Bunting, Daniel Silman, Minesh Karia, Sophie Johnson

**Affiliations:** Oxford Health Foundation Trust, Warneford Hospital, Oxford, UK; Oxford Health NHS Foundation Trust, Oxford, UK

**Keywords:** Antipsychotics, drug or substance interactions and side-effects, in-patient treatment, schizophrenia, dermatology

## Abstract

A patient developed fever, raised inflammatory markers and a maculopapular rash following commencement of clozapine for treatment of his schizoaffective disorder. Skin biopsy confirmed Sweet's syndrome. Identification of the cause was challenging, with a number of possible considerations including infection, malignancy and various potential drug triggers.

This case highlights the difficulties in the diagnosis of Sweet's syndrome, as well as in identifying the original trigger, which can have significant consequences for management. Withdrawal of potentially causative drugs must be balanced with their benefits, and decisions must be made in the best interests of the patient. Following two courses of prednisolone and withdrawal of clozapine, the patient's rash and systemic symptoms resolved. This confirmed the diagnosis of drug-induced Sweet's syndrome, with clozapine as the offending agent. His mental state stabilised on an alternative antipsychotic.

## Background

The case outlined here illustrates a rarely reported cutaneous reaction associated with clozapine use. Clozapine is an atypical antipsychotic, which is more effective than other antipsychotics for treatment-resistant schizophrenia,^1^ and acts primarily through binding to dopamine D2 receptors. However, it has a number of serious side-effects that limit its use as a first-line medication. The most significant of these is agranulocytosis, for which patients need regular monitoring. Other adverse effects include myocarditis, pancreatitis and seizures. Weight gain, insulin resistance and constipation are also common.

Skin reactions, including angioedema and pityriasis rosea, can occur in patients taking antipsychotics.^2^ Sweet's syndrome (or acute febrile neutrophilic dermatosis), first described in 1964,^1^ is an inflammatory disorder characterised by fever and raised tender erythematous skin lesions (papules, nodules or plaques). Lesion biopsy reveals a diffuse infiltrate of neutrophils, typically in the upper dermis, specifically the face, neck and upper extremities. Neutrophil count is usually elevated. Other symptoms include arthralgia, general malaise, headache and myalgia.

The underlying cause is uncertain, with hundreds of published cases. There are three main subtypes: classical (either associated with infections, inflammatory bowel disease and pregnancy, or idiopathic), malignancy associated (most commonly associated with acute myeloid leukaemia) or drug induced. A number of drugs have been linked to Sweet's syndrome, including granulocyte-colony stimulating factor. There have been two previous case reports of Sweet's syndrome associated with clozapine.^3,4^

For a diagnosis of drug induced Sweet's syndrome, five key criteria must be met:^5^

- abrupt onset of painful erythematous plaques or nodules;
- histopathologic evidence of a dense neutrophilic infiltrate, without evidence of leucocytoclastic vasculitis;
- pyrexia >38 °C;
- temporal relationship between drug ingestion and clinical presentation (or temporally related recurrence after oral challenge);
- temporally related resolution of lesions after drug withdrawal or treatment with systemic corticosteroids.

For classical or malignancy-induced Sweet's syndrome, criteria a and b plus two minor criteria must be met. These include:

- pyrexia >38 °C;
- association with an underlying hematologic or visceral malignancy, inflammatory disease or pregnancy, OR preceded by an upper respiratory or gastrointestinal infection or vaccination;
- a good response to treatment with systemic corticosteroids or potassium iodide;
- abnormal laboratory values at presentation (3/4): erythrocyte sedimentation rate >20 mm/h; positive C-reactive protein; >8000 leukocytes; >70% neutrophils.

The pathogenesis of Sweet's syndrome is not fully understood. The infiltrate is predominantly neutrophilic, with eosinophils also observed.^6^ In addition, oedema is usually present.

Management is primarily through systemic steroid therapy, typically oral prednisolone. Topical high-potency corticosteroids are used to treat localised lesions. Potassium iodide and colchicine are used as other first-line agents. Resolution can occur spontaneously, without therapeutic intervention. If the syndrome is drug induced, stopping the associated medication causes improvement and resolution. Restarting the offending drug is likely to result in recurrence. Recurrence is common in those with malignancy-associated Sweet's syndrome.

Of particular interest are the diagnostic and management challenges, especially when the causative agent is unclear. The risks to a patient's mental state from withdrawing medication – without certainty of its harms – while adding potentially destabilising effects of steroids must be considered.

## Case presentation

Mr B, a gentleman with a diagnosis of schizoaffective disorder, was admitted to an acute in-patient ward under the Mental Health Act owing to disinhibited behaviour. This second admission within a month represented a typical relapse pattern. Prior to this, he had been managed with a combination of amisulpride and promazine, avoiding admission for 10 years. Staff at his supported accommodation had become concerned about overfamiliarity. There were clear psychotic features including auditory hallucinations, bizarre ideas that he had died and was communicating with the deceased country singer Glen Campbell, grandiose proposals and delusional misidentification.

Settling Mr B's mental state with psychotropic medication proved difficult. In total, five antipsychotics (olanzapine, quetiapine, haloperidol, amisulpride and promazine) were trialled, with limited effect or poor tolerability. Overfamiliarity and chaotic behaviour continued to be core presenting features. For additional mood stabilisation, lithium was started. After escalation of the dose, and given the resistance of the patient's illness to other antipsychotics at this point, clozapine titration was commenced.

Subsequently, Mr B suffered a series of acute physical illnesses. On day 16 following initiation, he developed a fever with low blood oxygen saturation, not commensurate with his mild shortness of breath. He was admitted to a medical ward and diagnosed with acute bronchitis, with raised inflammatory markers but a normal chest x-ray. He was treated with intravenous co-amoxiclav, which was continued orally on medical discharge. Despite additional antibiotic cover with clarithromycin, he continued to experience pyrexia and tachycardia. Twenty-two days after clozapine initiation, a rash appeared, initially as papules on his upper arms then becoming more widespread. With ongoing derangement of his vital signs, he was transferred back to hospital for management on a medical ward, and dermatology opinion was sought. The rash had a maculopapular appearance and was pruritic and generalised, with confluent erythematous areas over the arms and trunk. Much of the rash was desquamating, with some plaques showing a punctum, and no obvious vesicles or blistering. Inflammatory markers remained elevated (white cell count 17.7 × 10^9^/L, C-reactive protein 92 mg/L).

A dermatological cause of his rash was considered. Severe cutaneous adverse reactions are reported in the literature for lithium,^7^ clozapine^2^ and amoxicillin-based antibiotics,^8^ all of which had been used. Obvious pustules and blistering were absent, ruling out a reaction on the Stevens–Johnson/toxic epidermal necrolysis spectrum.^9^ Maculopapular rashes are frequently observed as a non-immediate reaction to amoxicillin,^10^ which should resolve rapidly on discontinuation. The rash observed met some classic descriptions of DRESS (drug rash with eosinophilia and systemic symptoms) syndrome,^9^ though eosinophilia was absent. A skin biopsy performed by dermatologists confirmed a diagnosis of Sweet's syndrome.

A tapered course of prednisolone commenced 6 days after the onset of the rash resulted in resolution of the fever and rash over 2 weeks. This also coincided with a significant reduction in the patient's lithium dose owing to toxic plasma levels, as well as a modest reduction in clozapine dose owing to worsening liver function.

Given the patient's persisting psychotic features, lithium and clozapine were cautiously increased. However, Mr B suffered a further relapse of his rash 4–5 weeks following the first episode. This episode showed a more widespread eruption, associated with varying degrees of limb oedema. Inflammatory markers were again elevated. A second course of prednisolone was started, and dermatology recommended stopping lithium as the probable offending agent. However, it was clear that lithium was reliably stabilising the patient's mental state, and when it was reduced, he was more agitated, sexually disinhibited and banging on windows. Lithium was therefore not withdrawn and eventually further increased, as the steroid treatment continued.

During this second course of steroid treatment, the rash showed improvement but with persisting itch and papules. Inflammatory markers took 2 weeks to resolve. As a higher dose of lithium had not exacerbated the rash, the prevailing hypothesis was that clozapine had been the drug trigger for the patient's Sweet's syndrome. His psychotic complaints had not improved with clozapine. It was therefore slowly replaced with amisulpride. Two weeks into the clozapine-tapering regimen, Mr B's rash improved markedly and had completely resolved within a month. Following the reintroduction of amisulpride, his mental state also recovered significantly with no further delusional beliefs.

Once his mental state was stable, plans for discharge were made, with phased reintroduction to supported accommodation owing to his long admission. His social functioning and interaction recovered well. Following Mr B's discharge into the community, his mental state remained stable, and a subsequent reduction in his lithium (owing to tiredness) was the only alteration to his psychotropic medication. The Sweet's syndrome did not recur. Unfortunately, he has since contracted COVID and passed away.

## Discussion

Mr B's rash and physical deterioration raised an initial diagnostic conundrum, with the emerging possibility of a dermatological explanation for the ongoing fever supported by limited hard signs on chest examination. Subsequent biopsy was required for full confirmation of the diagnosis of Sweet's syndrome.

The cause of the Sweet's syndrome was the next diagnostic challenge. Classical Sweet's syndrome can be associated with infection. Although Mr B had been diagnosed with bronchitis prior to the development of his rash, both chest signs and chest X-ray changes were absent. The general malaise and fever may have been due to Sweet's syndrome. His laboratory abnormalities and pyrexia met the listed minor criteria for classical Sweet's syndrome.

During his admission, he experienced a pulmonary embolus, requiring anticoagulation. The co-occurrence of Sweet's syndrome and a thromboembolic event raised concern for an underlying malignancy. Incidental reactive inguinal nodes picked up when he underwent ultrasound of his leg provided further evidence. A computed tomography scan of the abdomen–pelvis did not identify any suspicious masses, and haematology were not concerned about risk of lymphoma or lymphoproliferative disorder, ruling out malignancy-induced Sweet's syndrome.

**Fig. 1:**
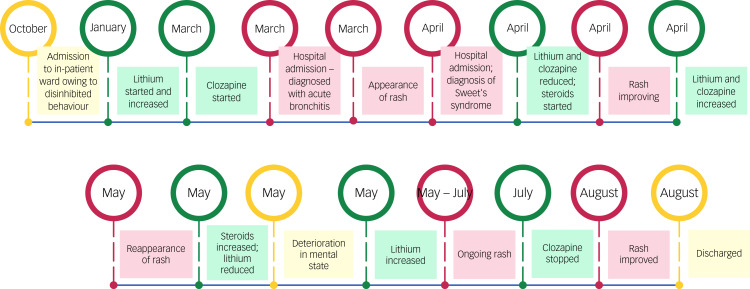
Timeline of the events described.

The next consideration was drug-induced Sweet's syndrome. Given recent increases in lithium and clozapine and the courses of antibiotics, identifying a possible drug trigger proved challenging. Consideration of the time course of the illness in relation to drug administration was the most helpful approach in confirming the diagnosis – the temporal criteria for drug-induced Sweet's syndrome were met in this case with respect to administration and withdrawal of clozapine (Fig. 1). According to the Naranjo algorithm adverse drug reaction probability scale, the link between clozapine and Sweet's syndrome was probable (a score of 6).^11^

The literature regarding the association of clozapine and Sweet's syndrome is limited, with only two published case reports. This case report adds to the evidence that Sweet's syndrome may be caused by clozapine; prescribers should be aware of this.

The pathogenesis of Sweet's syndrome has not been definitively determined, with suggestions that cytokines might have an aetiological role. There is evidence of clozapine-induced fever being mediated by pyrogenic cytokines.^12^ Findings of transient increases in plasma G-CSF levels have been observed with clozapine and demonstrated to be most prominent by the end of the second week of treatment, with increases in levels of other cytokines and receptors.^13^ Notably, emergence of clinical symptoms within 2 weeks of commencement has been reported in the previous literature and occurred in this case. Further research is required to establish whether the association between Sweet's syndrome and clozapine is driven by the influence of cytokines, or whether an alternative mechanism can be identified.

## Conclusions

This case highlights the difficulties in clinical practice of definitively determining which subtype of Sweet's syndrome a patient is experiencing. The varied and multi-organ extra-cutaneous manifestations of Sweet's syndrome, the adverse effects associated with clozapine and the use of other medications in the treatment of mental illness can further complicate clear identification of a single causative agent of Sweet's syndrome.

With drug induced Sweet's syndrome, treatment includes cessation of offending drug, as well as steroid treatment, which has known mood destabilising properties. There is often difficulty in identification, given the polypharmacy that many psychiatric patients experience. The best management plan may be selective withdrawal of potential candidates with monitoring of clinical outcomes. In complex patients, the risk of withdrawal of possible causative drugs must also be considered, so treatment plans should be individualised.

## Consent statement

Written and verbal consent was obtained from the next of kin of the patient described in this report.
